# Incidental Detection of Post-cholecystectomy Lemmel Syndrome With Burr Hole Plating and Ventriculoperitoneal Shunt: A Cadaveric Case Report of Quadruple Pathology and Clinico-Pathophysiological Correlation

**DOI:** 10.7759/cureus.91911

**Published:** 2025-09-09

**Authors:** Sanjoy Sanyal, Marie Affana, Adedolapo A Adegbite, Oluwaseun D Amodu, Veronica K Ayiku

**Affiliations:** 1 Anatomical Sciences, All Saints University School of Medicine, Roseau, DMA

**Keywords:** cadaver case report, cranial burr hole and plating, duodenal diverticula, lemmel syndrome, medical education, periampullary duodenal diverticulum, positive unintended consequences, post-cholecystectomy, subdural hematoma, ventriculoperitoneal shunt surgery

## Abstract

During surgical anatomy dissection on a cadaveric subject for educational purposes in a medical university, we encountered a rare syndrome and evidence of a constellation of three past surgeries, which provided us with an intellectually rewarding tapestry of clinical and academic findings sufficient to enrich the overall learning experience. During her lifetime, the elderly female subject had undergone a cholecystectomy, presumably for calculous cholecystitis. The dissection also revealed what was putatively diagnosed as type III Lemmel syndrome, characterized by a large periampullary duodenal diverticulum and dilated common bile duct, apart from a smaller duodenal diverticulum near the duodenojejunal flexure. She had a ventriculoperitoneal (VP) shunt implanted from the occipital horn of the right ventricle to the right hypochondrial peritoneal cavity. Finally, she had a right parietal skull burr hole covered with a titanium cranial implant after the evacuation of a possible subdural hematoma during her lifetime. No description could be found in the cadaveric dissection literature with this constellation of findings in one subject. There is a significant relationship between duodenal diverticula and gallstone disease, especially choledocholithiasis. The possible interplay between the two intracranial findings is complex and can be bidirectional, which are discussed in this case report. There is no known clinico-pathophysiological relationship between biliary tract and duodenal findings, on the one hand, and the intracranial observations, on the other. However, a possible association between intra-abdominal pathology and VP shunt malfunction has been postulated. This case report is also an expression of the law of unintended consequences with positive effects. What was supposed to be a routine cadaveric dissection for anatomical studies turned out to be a veritable clinico-pathophysiological discourse. Instead of merely restricting our medical teaching to normal anatomical findings in cadaveric dissections, we adopted an open-minded approach to enrich the learning experience for not only entry-level medical students but also higher-level medicos and even surgical residents. This opened up hitherto overlooked pathophysiological vistas that enabled us to exercise our clinico-pathophysiological acumen, as is demonstrated in this case report.

## Introduction

While performing a routine cadaveric dissection on an elderly female subject, for teaching Gross Anatomy to MD-1 students in a medical university, the principal author encountered a rare syndrome and a constellation of three past surgical interventions that had been performed during the subject’s lifetime. The rare pathology under tentative consideration was possibly Lemmel syndrome. Gerhard Lemmel in 1934 described a large periampullary duodenal diverticulum (PADD) compressing the ampulla of Vater to obstruct both the common bile duct (CBD) and pancreatic duct, leading to obstructive jaundice. Only 17 such cases have been confirmed to date in live subjects, with a 60% female preponderance [1]. The three past surgical interventions were (A) a laparoscopic cholecystectomy, presumably for calculous cholecystitis, likely complicated by choledocholithiasis; (B) a ventriculoperitoneal (VP) shunt placement; and (C) a right parietal skull burr hole surgery, covered with a titanium cranial implant, possibly for subdural hematoma (SDH) [2,3].

While the three surgical interventions are not uncommon in living subjects, no description could be found in the anatomical dissection literature with all these findings in a single cadaveric subject. With minimal antecedent data available and no antemortem imaging or clinical findings to substantiate the diagnosis, it was an academic exercise to try to reconcile the disparate pathological findings in the subject and establish a clinico-pathophysiological correlation between them to arrive at a cogent conclusion as to what transpired during the subject’s lifetime. This constituted the first objective of this case report. The relationship between biliary and duodenal findings is well established, and the literature is rife with such associations, spanning several decades [4,5]. The possible interplay between the two intracranial procedural findings is complex and bidirectional, which is discussed in this case report [6,7]. There is no known direct relationship between the biliary tract and duodenal findings, on the one hand, and the intracranial observations, on the other. However, a possible relationship between intra-abdominal surgery and/or pathology and consequent VP shunt malfunction has been postulated in this case report [8].

The second objective underpinning this case report was to highlight the positive effects of the law of unintended consequences in medical education [9]. While performing cadaveric dissections in medical institutions for educational purposes, being cognizant of unexpected incidental pathologies and evidence of past surgeries in the subjects, and incorporating them in the teaching of not only entry-level MD students but higher-level medical students, and even surgical residents, went a long way toward making them into holistic clinicians, while honing the clinical acumen of all concerned.

## Case presentation

An elderly female cadaver subject, wrapped in a linen sheet saturated with 37% formalin and 5% of 85% phenol disinfectant, and sealed in a 6Mil (6/1,000th inch) low-density polyethylene bag, was procured from a licensed embalmer from New Jersey, USA, after it was approved for release by the latter state government. The body had been embalmed through the right common carotid artery with 10 gallons of a solution consisting of 25% of 85% phenol solution, 25% of diethylene glycol, 25% of water, and 25% of alcohol (one-third methanol and two-thirds ethanol). To this mixed solution, 3 ounces of formalin were added to each 3 gallons of solution. The embalming process was undertaken by a duly licensed agency that was certified to practice embalming by the Louisiana State Board of Embalming and Undertaking.

The method of disposition of the deceased subject was explicitly stated as “donation.” The cadaver was a donated non-clinical human tissue on loan from US medical schools, with written permission for it to be exported for medical research and education purposes to our university medical college. It was incumbent on the borrowing school to ensure the dissected remains were exported back to the lending institution in the United States for cremation under government regulations stipulated in the 1966 Pan American Health Organization (PAHO) XVII Resolutions. A donor form permitting the executor of the deceased is on file at the lending institution in the United States. This form may not be released to anyone because of the Health Insurance Portability and Accountability (HIPAA) Law, which was enacted in the United States to give privacy to all patients’ medical information.

As per legally disclosed information at the receiving institution, at the end of her life in December 2016, the subject was an 88-year-old Caucasian female who was certified as being free from all communicable diseases. During her lifetime, the subject was high school educated or GED completed, a secretary by vocation, and widowed. The immediate cause of death was mentioned only as “cardiopulmonary arrest.” The manner of death was certified as “natural.”

Our medical institutional curriculum is divided into three semesters each year, with 15 weeks in each semester. Each 15-week semester is further subdivided into three blocks of five weeks each, referred to as Blocks 1, 2, and 3. Gross Anatomy is taught in the first semester (MD-1) of the MD program. Therefore, the surgical anatomy cadaveric dissections are also performed during MD-1 teaching, and are divided into three blocks. Abdominal and thoracic dissections are performed during the five-week period of Block 2, and cranial and cervical dissections are performed during the five-week period of Block 3.

**Figure 1:**
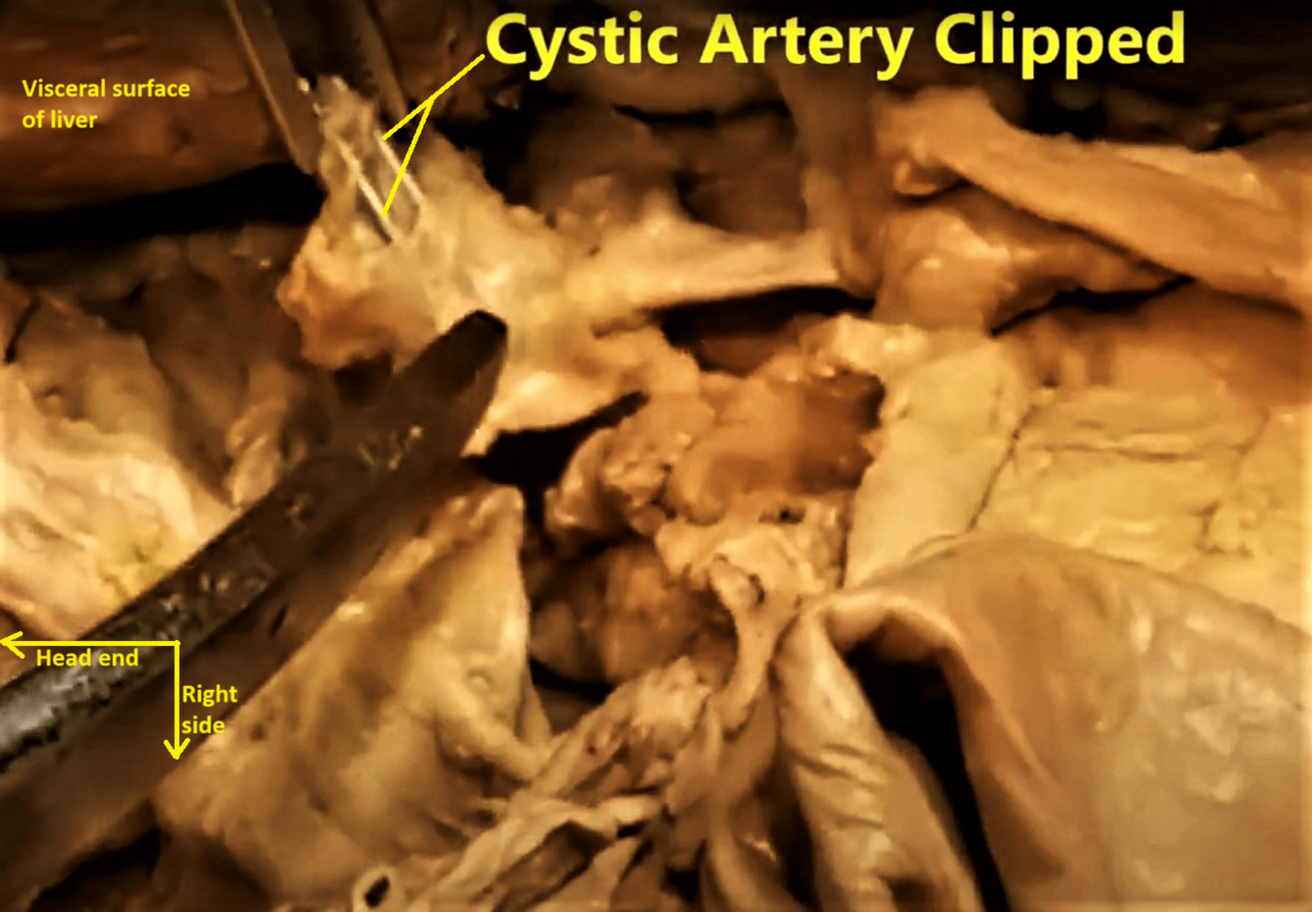
Enlarged view of the region of Calot’s triangle, as visualized during abdominal dissection by the principal author. The instrument in the lower left quadrant is pointing at the region of Calot’s triangle (what was left of it). The double-pronged thumb forceps in the upper left quadrant is picking up the double-clipped pedicle composed of the fibrosed cut ends of the cystic artery and cystic duct.

On general inspection, the embalmed cadaver was well-preserved, with no noteworthy external findings. The deceased subject herself could be described as slim in build, with an asthenic body type. While performing thoraco-abdominal dissections during the Block 2 period of the MD-1 program, a white Teflon tube was noticed entering through the right upper anterior abdominal wall into the abdominal cavity. There were no other visible scars on the abdominal wall, surgical or otherwise. While dissecting inside the abdomen, on attempting to trace the cystic artery from the right branch of the hepatic artery proper, a noticeable double-metallic-clipped fibrosed structure was visualized, but no cystic artery (Figure 1).

**Figure 2:**
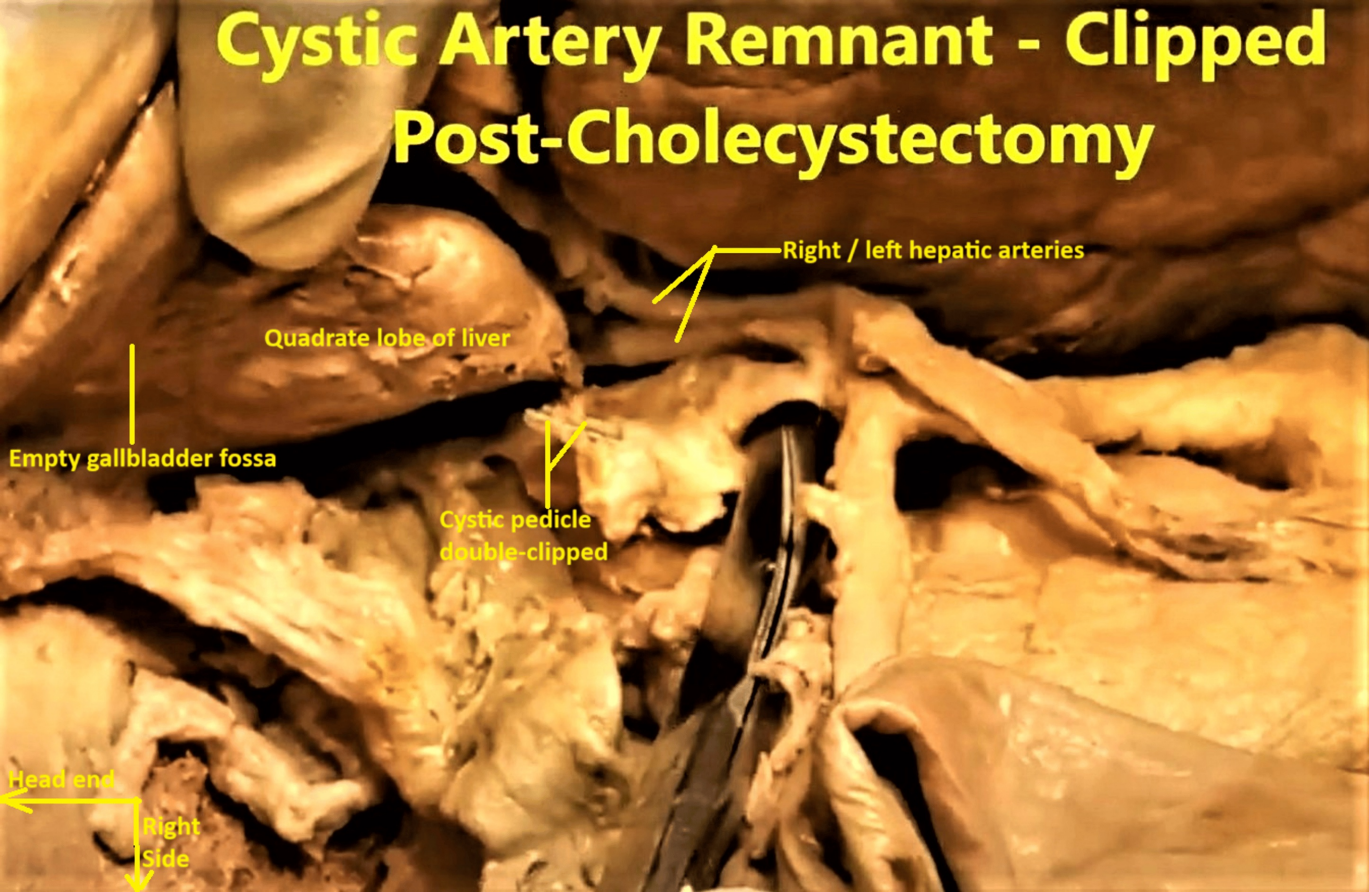
Zoomed-out view of the region of Calot’s triangle. The double-clipped cystic artery-duct pedicle and the empty gallbladder fossa next to quadrate lobe of liver is visible, testifying to the post-cholecystectomy status of the subject.

Detailed checking revealed that the region of what was supposed to be Calot’s triangle was fibrosed, its inferior boundary was missing, the fibrosed stump of the cystic duct was included in the double-clips, and the gallbladder fossa was empty next to the quadrate lobe of the liver (Figure 2). As there was no visible laparotomy or Kocher’s subcostal incision scar on the abdominal wall, these findings were construed to mean the subject had undergone laparoscopic cholecystectomy during her lifetime.

**Figure 3:**
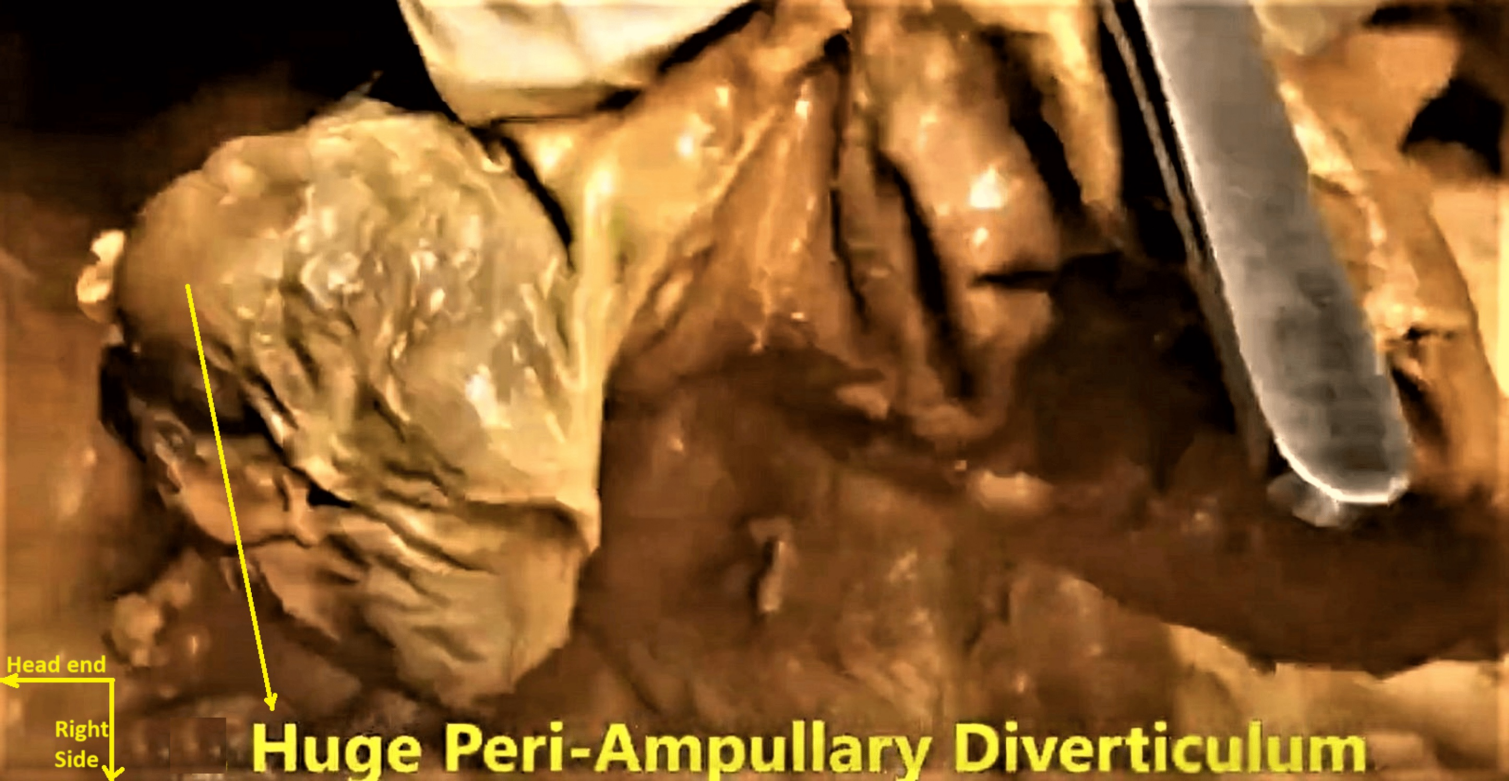
Huge periampullary duodenal diverticulum arising from the descending (D2) part of the duodenum. This was delineated while dissecting in the region of the C-loop of the duodenum and the head of the pancreas.

**Figure 4:**
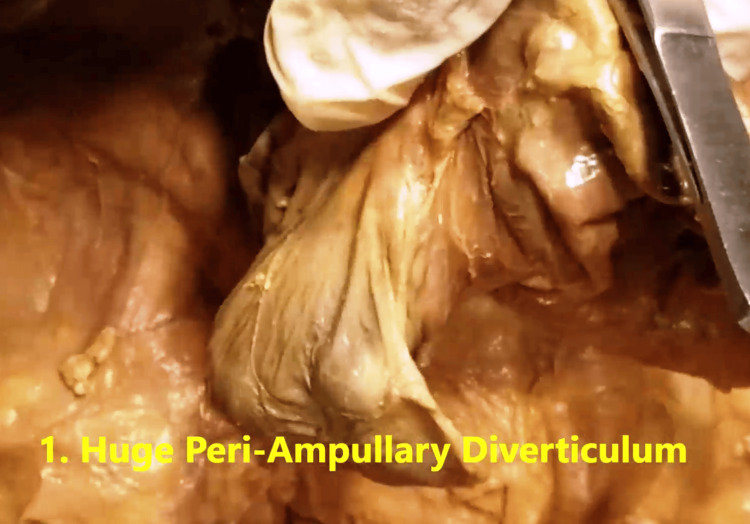
Another view of the periampullary duodenal diverticulum arising from posteromedial wall of the D2 part of the duodenum. The duodenum has been dissected off from the posterior abdominal wall and picked up between the thumb and index finger by the principal author, showing the periampullary duodenal diverticulum hanging down.

While dissecting in the region of the C-loop of the duodenum and the head of the pancreas, a large PADD was identified in its second (descending) part, D2, arising from its posteromedial wall (Figures 3, 4).

**Figure 5:**
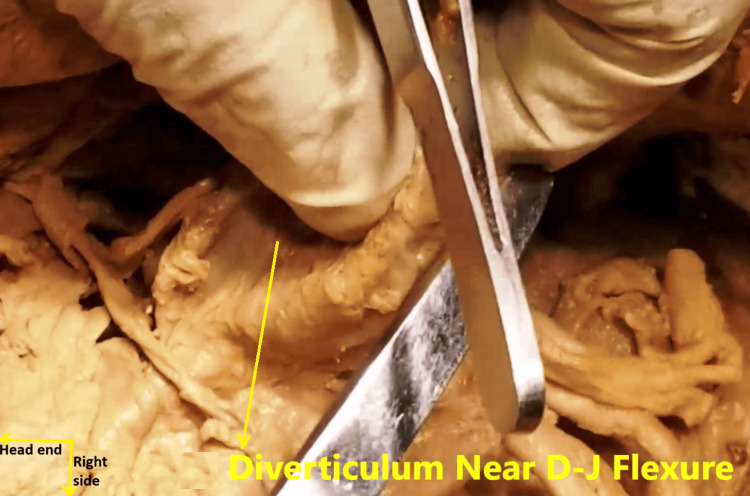
A smaller diverticulum arising from the ascending (D4) part of the duodenum, near the duodenojejunal (D-J) flexure. This has been picked up between the thumb and index finger by the principal author.

**Figure 6:**
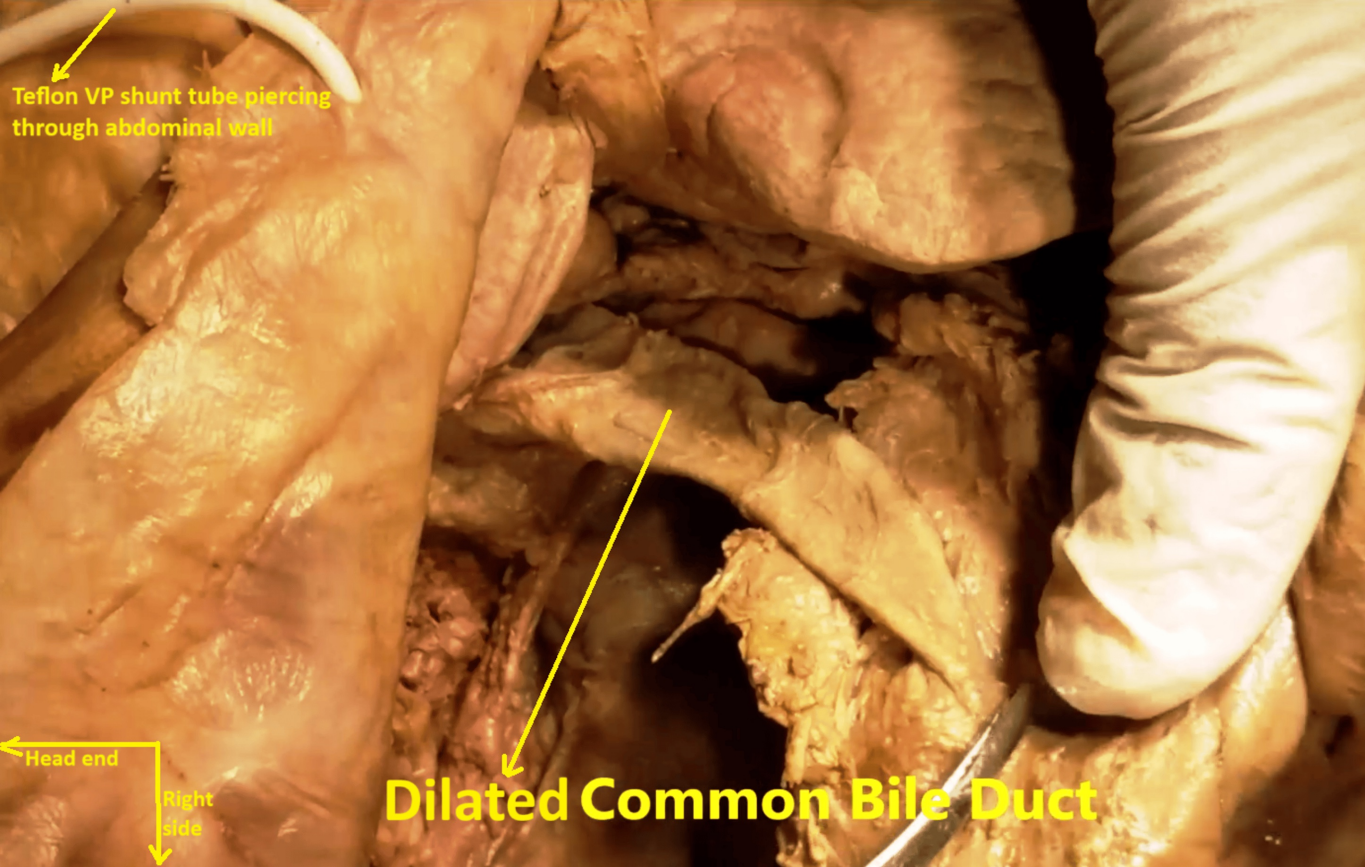
Supra-duodenal part and part of the intra-pancreatic part of the common bile duct. The common bile duct was moderately dilated to about 8 mm. Incidentally, the Teflon ventriculoperitoneal shunt tube, described later, is visualized piercing through the abdominal wall in the upper left quadrant of the image.

A second, smaller diverticulum was delineated in the fourth (ascending) part of the duodenum, D4, near the duodenojejunal flexure (Figure 5). The CBD was found to be moderately dilated to about 8 mm (Figure 6). Given this set of findings, it was tentatively diagnosed that the subject had Lemmel syndrome, which most probably went unnoticed during her lifetime [1].

**Figure 7:**
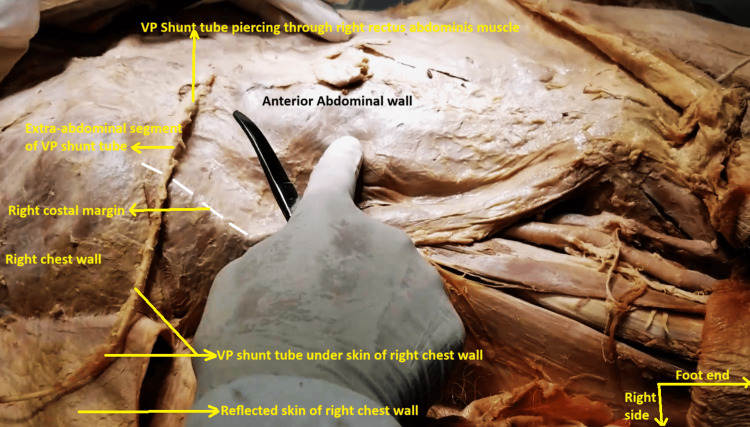
Subcutaneous part of the ventriculoperitoneal shunt tube over the right chest and abdominal wall. The skin of the right lower chest wall and abdominal wall has been dissected off. This part of the ventriculoperitoneal tube is encased in fibrous tissue. It is seen piercing through the right anterior rectus sheath and right rectus abdominis muscle.

**Figure 8:**
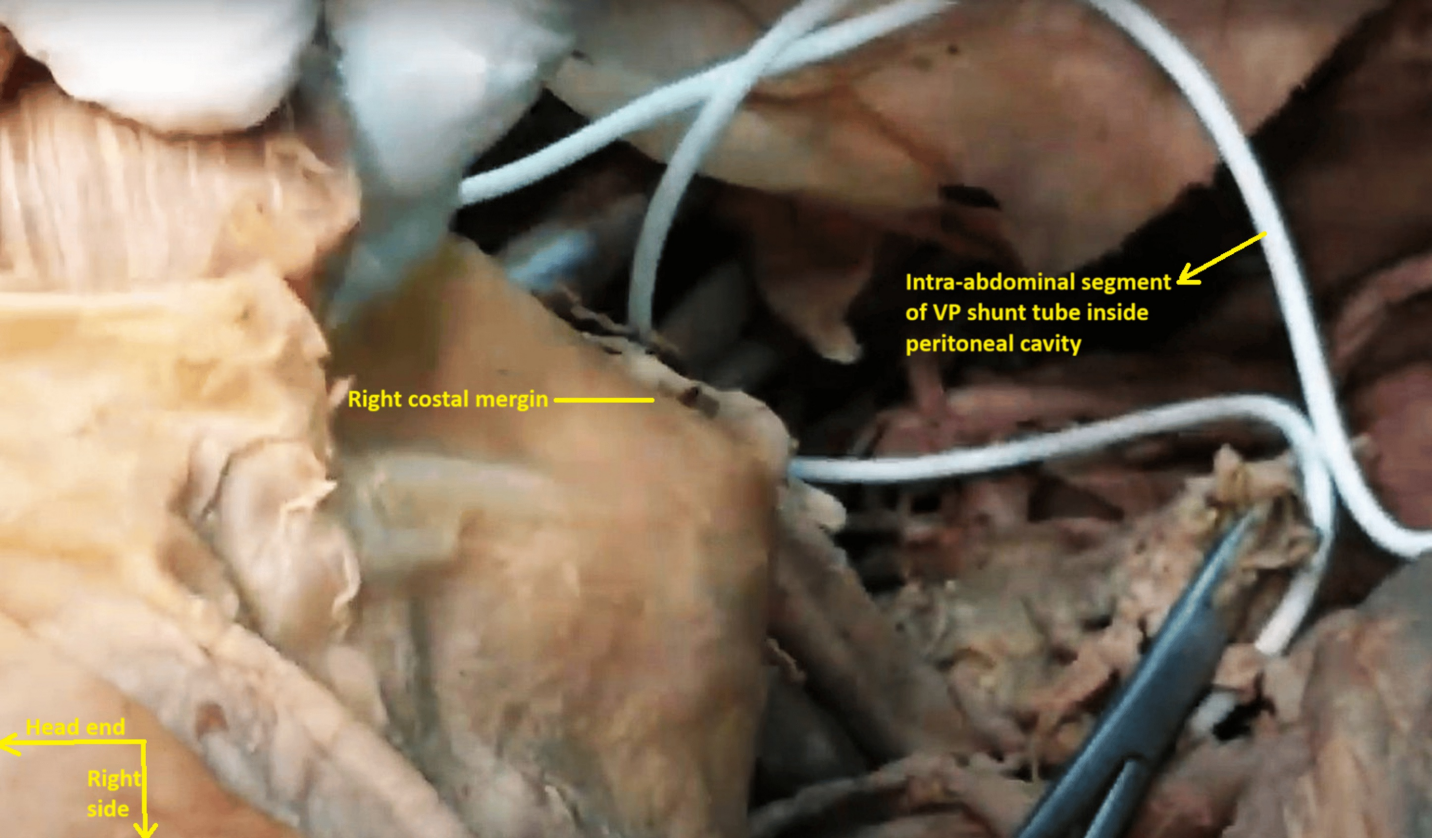
Intra-abdominal segment of the ventriculoperitoneal shunt tube can be seen coiled under the right costal margin. It is in the right subhepatic space inside the right hypochondrial peritoneal cavity. Note the pure white color of the intra-abdominal segment of the tube and compare it with the dirty brown, fibrous tissue-covered subcutaneous part of the same tube in the previous image.

On the abdominal wall itself, a white Teflon tube was found traveling subcutaneously from across the right costal margin and piercing through the right rectus sheath and rectus abdominis muscle and entering the abdominal cavity. While the extra-abdominal segment was covered by dirty brown fibrous tissue, the intra-abdominal segment was pristine white and coiled under the right costal margin. It was opening inside the subhepatic space in the right hypochondrial peritoneal cavity (Figures 7, 8).

**Figure 9:**
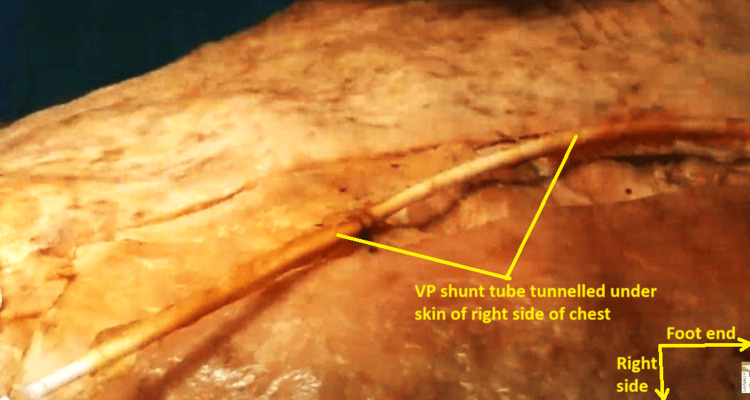
Fibrous tissue-covered ventriculoperitoneal shunt tube in the subcutaneous plane of the right chest wall. The skin of the right side of the chest wall has been dissected off to show the tube.

**Figure 10:**
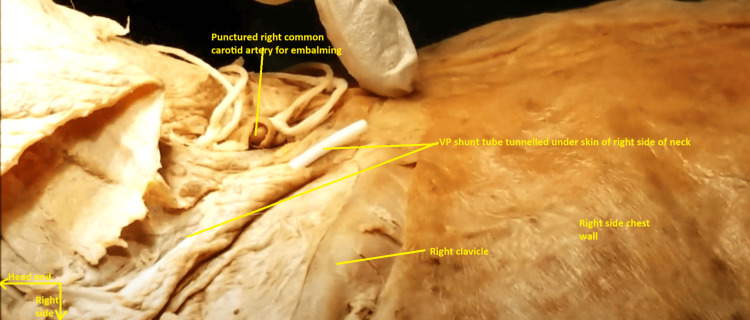
Ventriculoperitoneal shunt tube in the subcutaneous plane of the right side of the neck. The skin of the right side of the neck has been dissected off. Incidentally, the right common carotid artery can be visualized further medially in this image. It was punctured for embalming purposes, with ligatures placed proximal and distal to the puncture site.

This tube was traced retrogradely, and it was noticed that it had been tunneled under the skin of the right side of the chest wall, up to the right side of the neck. The entire subcutaneous segment of the tube across the right chest wall and over the right abdomen was encased in dense fibrous tissue and dirty brown in color (Figures 9, 10).

**Figure 11:**
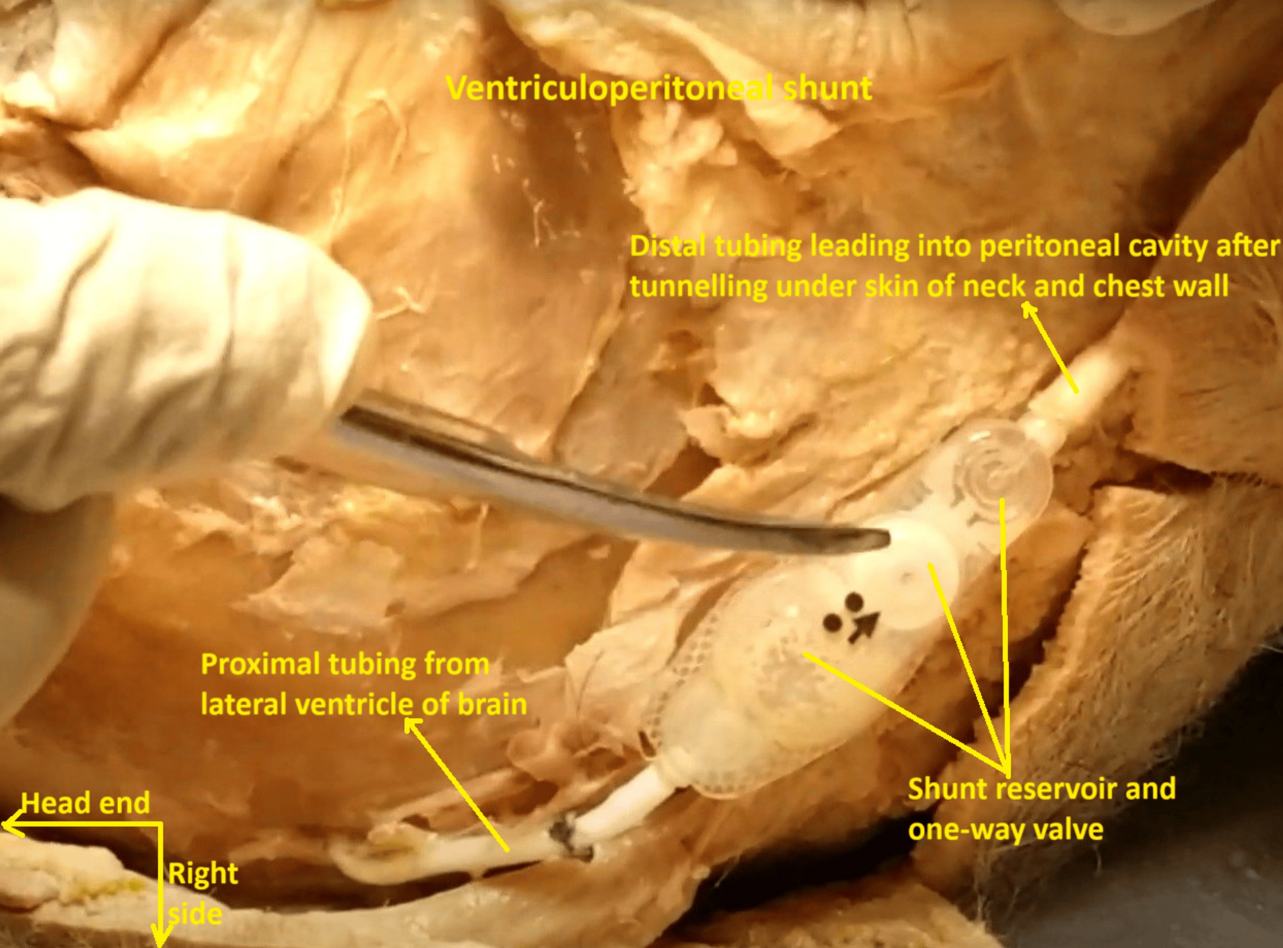
Ventriculoperitoneal shunt reservoir (with its contained pressure-regulated valve) is clearly visible. This image was captured during a right parieto-occipital scalp dissection of the same cadaveric subject. The proximal tubing is seen emerging from the right occipital skull. The distal tubing was leading down into the subcutaneous tissues of the neck, as shown in Figure 10.

During Block 3 of the same semester (MD-1), the head and neck regions came up for dissection in the same cadaveric subject, as part of our usual Gross Anatomy curriculum. When the scalp proper was dissected off the skull, a stump of a Teflon tube was noticed emerging from the right occipital skull. It was a straight non-flanged tube with multiple apertures near the catheter tip, which was meant to drain cerebrospinal fluid (CSF) from the lateral ventricle. It led to a reservoir with a one-way valve under the right side of the scalp (Figure 11).

Distally, the reservoir led into another Teflon tube, which was tunneled under the skin of the right side of the neck and right side of the chest (Figures 9, 10). This was found to be continuous with the aforementioned Teflon tube that was traced retrogradely up from the right hypochondrial cavity (Figures 7, 8). Judging by its origin and observed location, most probably it was leading from the occipital horn of the right ventricle to the right peritoneal cavity, greater sac. It was posited that a VP shunt had been implanted during the subject’s lifetime. Judging by its appearance, it appeared to be a pressure-regulated VP shunt-valve system [2,7].

**Figure 12:**
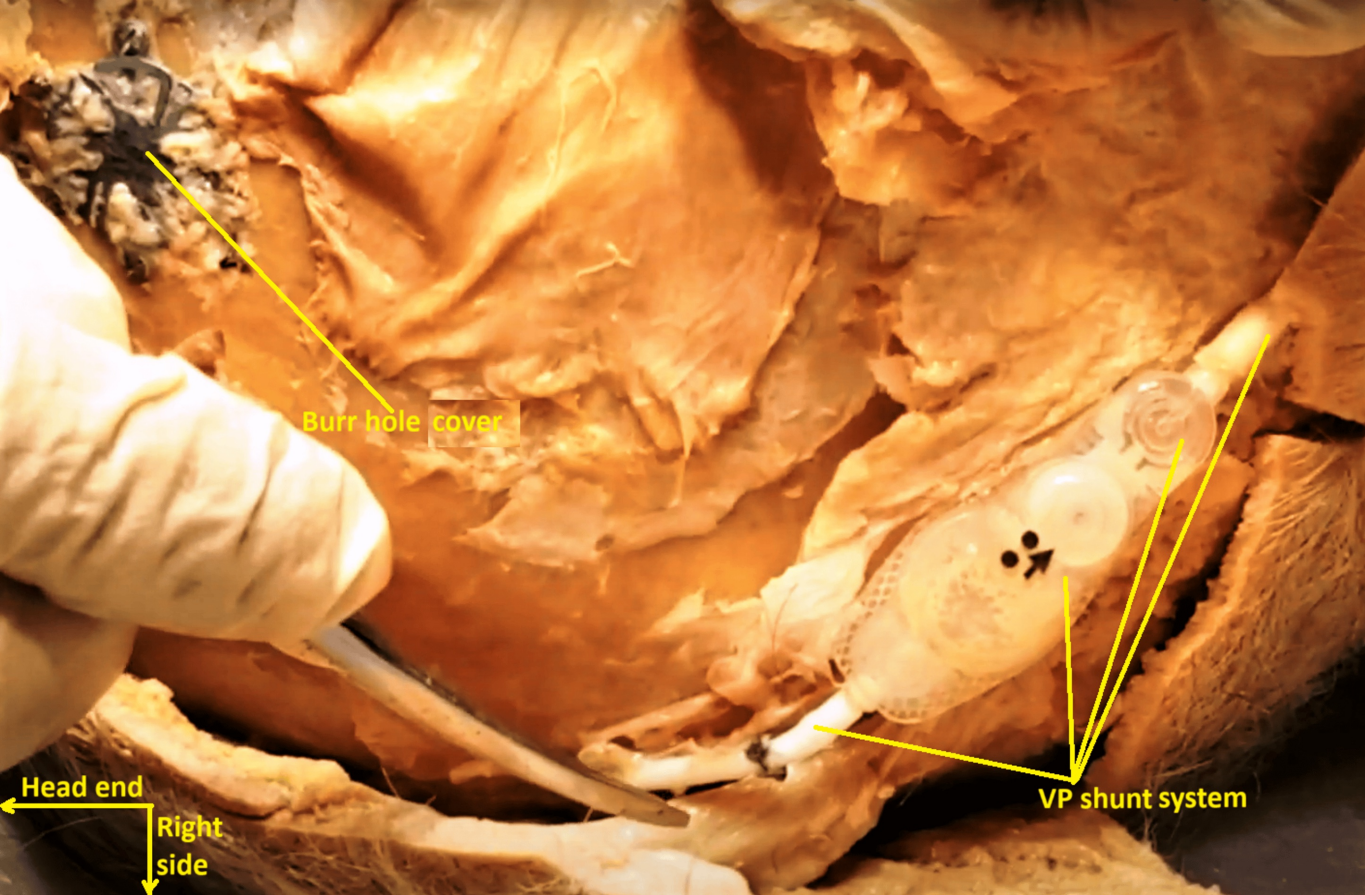
Medially directed view of the same dissection sequence to show the ventriculoperitoneal shunt system and cranial plated burr hole. The ventriculoperitoneal shunt system is seen in the right lower quadrant of the image. The cranial-plated burr hole on the right parietal skull is in the left upper quadrant of the image.

**Figure 13:**
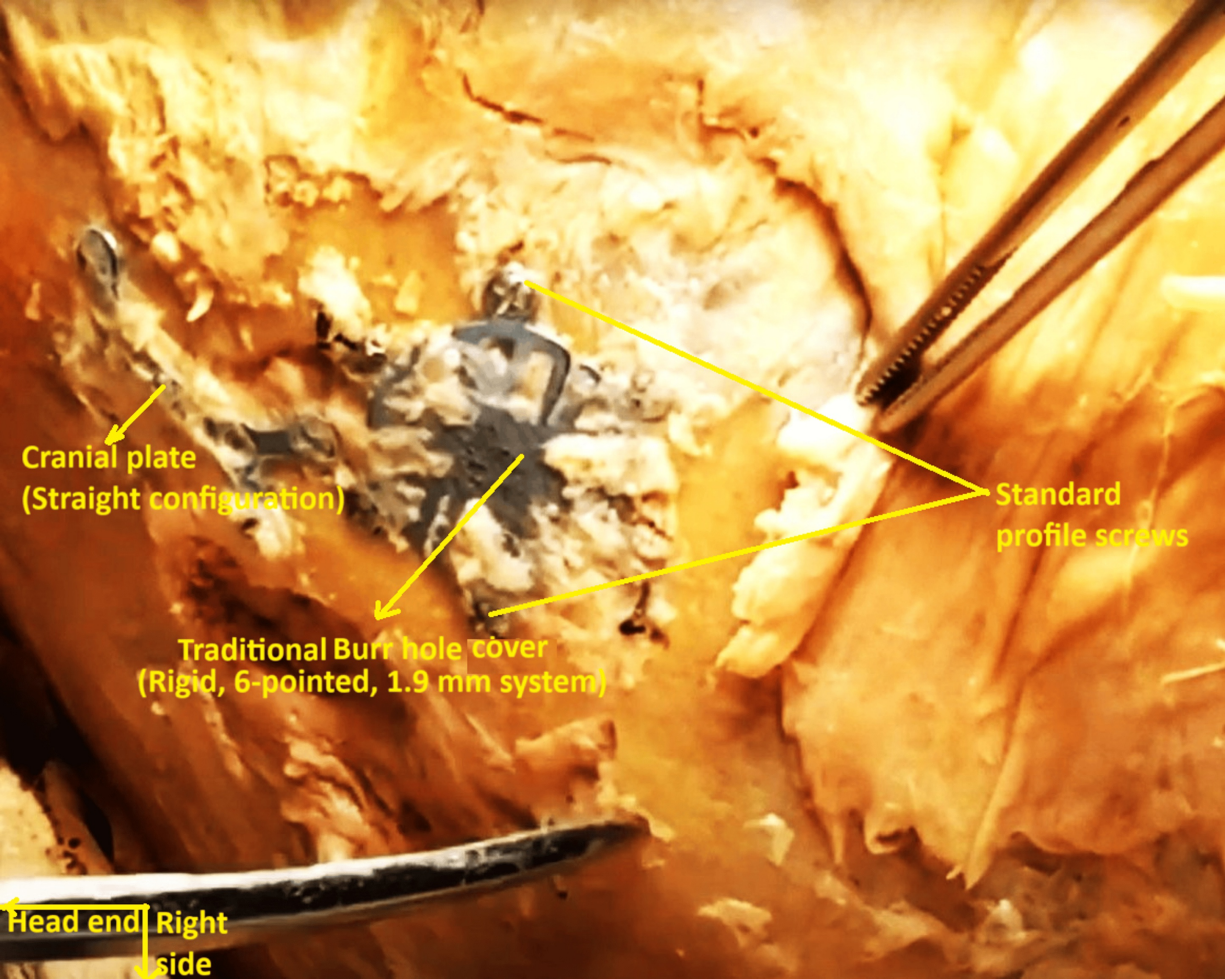
Localized view of the cranial-plated burr hole region of the right parietal skull. The burr hole was covered by a circular cranial plate, made of titanium alloy, fitted with six peripheral screws, and a single separate straight cranial cross-plate.

Medial to the proximal end of the VP shunt tube emerging from the right occipital skull, remnants of a surgical 1.9 mm burr hole, encased in dense fibrous tissue, were detected in the right parietal skull (Figure 12). It was covered by a circular cranial plate, possibly made of titanium alloy, fitted with six peripheral screws, and a separate single straight cranial cross-plate (Figure 13). The former conformed to the description of a traditional, rigid burr hole cover, 1.9 mm system [3].

Thus, it was surmised that the subject had a burr hole surgery performed on her skull during her lifetime. There were no findings to substantiate the cause-and-effect relationship between the VP shunt implant or the burr hole surgery. Neither was there any evidence in the dissected specimens to delineate the temporal sequence of the two surgeries.

All dissected parts were sprayed with proprietary Carolinas Perfect Solution® (www.carolina.com) to preserve the tissues, prevent desiccation, and enable high-contrast image acquisition. Using a dual iPhone 13 camera, with 12-megapixel, f/1.6, 26 mm (wide), 1.7 μm, dual pixel phase-detection autofocus features, and sensor-shift optical image stabilization, 12 MP, f/2.4, 13mm, 120° (ultrawide) capabilities, all findings were graphically documented in landscape mode in high resolution and contrast. This enabled display and demonstration to not only MD-1 Gross Anatomy students but also higher-level MD students and even surgical residents for educational purposes.

## Discussion

No description could be found in the dissection literature of the constellation of findings described in this single cadaveric subject. There is a significant relationship between duodenal diverticula and choledocholithiasis [4,5]. The possible interplay between the two intracranial findings, namely, VP shunt and cranial plate-covered burr hole, is complex and possibly bidirectional [6-8]. There is no known pathophysiological relationship between biliary and duodenal findings, on the one hand, and the intracranial observations, on the other. However, a possible association was postulated later, based on available observations [8].

Duodenal diverticula and biliary lithiasis

Over the last several decades, several compelling associations have been described in the literature between duodenal diverticula, as was seen in our subject, and biliary lithiasis. However, the exact cause-and-effect relationship has not always been intuitive [4,5]. Patients with duodenal diverticula have a higher frequency of gallstone disease and recurrent bile duct stones after biliary surgery or endoscopic sphincterotomy, compared to non-diverticular patients [10-13]. Duodenal diverticula can influence bile duct diameter, even in the absence of bile duct stones. They may chronically stimulate the papilla of Vater, produce bile stasis, bacterial contamination of the bile, and biliary tract infection, leading to the formation of gallstones and choledocholithiasis [1]. These stones are often bilirubinate stones, though superimposed infection can lead to calcium deposition [10-13]. Lemmel syndrome is a rare cause of obstructive jaundice caused by a PADD compressing the intra-pancreatic CBD [1].

Lemmel syndrome

The classical Lemmel syndrome, as described by its original author, Gerhard Lemmel, in 1934, stipulated that the PADD has to be large or significant enough to compress the ampulla of Vater to obstruct both the CBD and pancreatic duct, leading to obstructive jaundice [1]. It is a rare diagnosis; only 17 confirmed cases have been found in the literature after excluding all ambiguities [1]. However, others have suggested that Lemmel syndrome should be considered in all cases of suspected pancreato-biliary disorders [14]. Yet others have posited that pancreatic involvement is not a *sine qua non *of Lemmel syndrome [1]. Though ultrasonography, CT, and MRI have been advocated for its diagnosis, endoscopic retrograde cholangiopancreatography (ERCP) has been claimed to be the gold standard diagnostic test for Lemmel syndrome [1,14]. Lemmel syndrome has been classified as Type I (major duodenal papilla within the duodenal diverticulum), Type II (major duodenal papilla in the margin of the duodenal diverticulum), and Type III (major duodenal papilla near the duodenal diverticulum) [1].

Based on this classification, Type III Lemmel syndrome was a postmortem diagnosis made by us in our cadaveric subject. The large PADD was near the major duodenal papilla, with a dilated CBD, though there was no antemortem mention or evidence of icterus in our subject. The CBD dilatation could have been due to compression by the large PADD. In the absence of ERCP findings, the status of the pancreatic duct could not be elucidated. The fact that cholecystectomy had been performed during the subject’s lifetime, the most common indication for such a surgery being gallstone disease and cholecystitis, and the fact that there is a strong association between duodenal diverticular disease and biliary lithiasis, adds credence to our diagnosis. While CBD dilatation may be a postoperative change often seen after cholecystectomy, it may also be a result of exploration for past choledocholithiasis [4,5,10-13]. In all probability, Lemmel syndrome was never diagnosed during the subject’s lifetime because of the rarity of the condition from the average clinician’s perspective. Perhaps a greater index of clinical suspicion in all cases of pancreato-biliary disease, followed by investigation of all such cases with ultrasound, CT, MRI, and ERCP, may lead to more cases of Lemmel syndrome being diagnosed than is hitherto the case [14].

Ventriculoperitoneal shunt

The clinical conundrum that begged resolution in our subject was the indication for her VP shunt insertion from the occipital horn of the right ventricle to the right peritoneal cavity. The literature is quite categorical in its assertion that the incidence of aneurysmal subarachnoid hemorrhage (SAH) is 1.2 to 2 times higher in females than in males. Women tend to be older at the time of diagnosis, and the average age of females diagnosed with SAH is higher than males [15]. Our subject tends to fit this demographic entity eminently. SAH, *per se*, or complicated by intraventricular hemorrhage, can lead to blockage of CSF absorption through arachnoid villi, leading to increased intracranial pressure (ICP) and hydrocephalus. In such situations, a VP shunt is a viable surgical option to alleviate the increased ICP [16].

Most VP shunt valves in use are differential pressure-regulated, as was noticed in our subject. These devices are designed to open if the pressure difference across the valve exceeds a predetermined value. This value may be set in advance, or if the newer programmable valves are being used, they may be regulated afterwards from outside. As the pressure across the valve increases, the resistance decreases. This permits as much CSF to flow as is required to reduce the ICP closer to the opening pressure. Postural over-drainage symptoms are common in these types of VP shunt valve systems, especially during standing, due to the so-called “siphon effect” from the abdomen. This may be averted to some extent using anti-siphon devices to minimize flow in the erect posture [2,6-8,16,17].

VP shunt valves may also be flow-regulated (as in Orbis-Sigma valve shunt), though this was not incorporated in our subject. In these shunt systems, CSF flows from the ventricles toward the peritoneal cavity via a valvular device with a diaphragmatic aperture. As the flow rate rises above 20 mL/hour, the diameter of the diaphragmatic aperture decreases, increasing the resistance of the valve and thereby regulating flow. Countering this mechanism, a safety mechanism is also incorporated that reduces the resistance at differential pressures of 25-30 mmHg. This alleviates precipitous elevations in intracranial pressure [2,7,17].

VP shunt function is determined by resistance to CSF flow and CSF pressure. These two parameters are governed by two mathematical equations that can be partially tested experimentally. One formula measures resistance to CSF outflow (R^_[CSF]_^) by the arachnoid villi after infusing fluid into the lumbar cistern at a steady rate (infusion rate (IR)), and helps to predict shunt performance. This is analogous to Ohm’s law of electrical resistance, and is summarized in Equation 1 below [2,7]:

\begin{equation} R_{\text{CSF}} = \frac{\text{Pressure}_{\text{Plateau}} - \text{Pressure}_{\text{Baseline}}}{\text{IR}} \label{eq:rcsf} \end{equation}

The other formula measures the CSF draining pressure (DP) in VP shunts by summation of ICP, hydrostatic pressure (HSP), which itself is a function of CSF density and vertical height of the fluid column, and intra-abdominal pressure (IAP), as shown in Equation 2 below. It predicts CSF overdrainage (siphon-effect) or underdrainage in VP shunts [8]:

\begin{equation} DP = ICP + HSP - IAP \label{eq:dp_equation} \end{equation}

**Figure 14:**
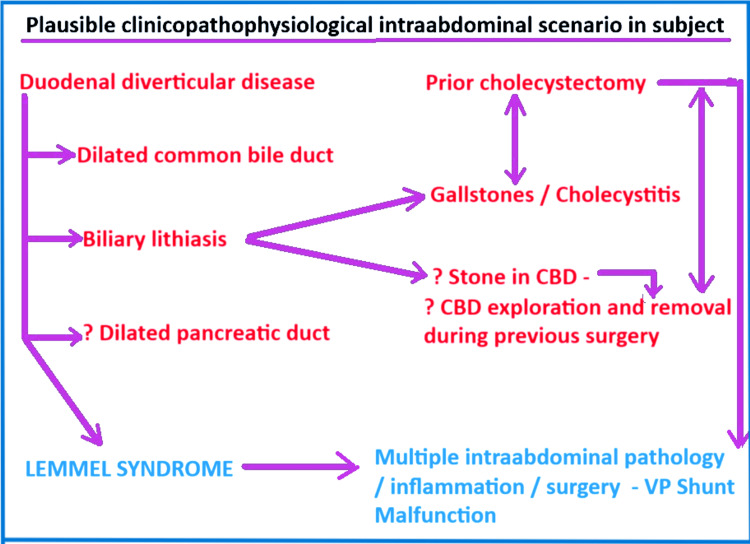
Schematic flow diagram demonstrating the postulated clinico-pathophysiological scenario in our subject. The flow diagram attempts to consolidate the biliary and duodenal findings into the rarely described entity called Lemmel syndrome. It also attempts to explain how intra-abdominal findings may have contributed to VP shunt malfunction. Image created by the principal author, assisted by Ms. Pritam Shahukar of All Saints University School of Medicine. It was created on MS Paint and enhanced using the Windows 11 Photos App. CBD = common bile duct; VP = ventriculoperitoneal

VP shunts are more prone to malfunction in increased IAP states (obesity, high body mass index (BMI), chronic constipation, chronic urinary retention, past abdominal surgery, and bowel inflammation) [8]. In our subject, there was evidence of intra-abdominal inflammation (possibly past cholecystitis), pathology (duodenal diverticula, dilated CBD), and past abdominal surgery (cholecystectomy), all of which could have contributed to VP shunt malfunction. The schematic flow diagram in Figure 14 provides a possible scenario in our subject that could connect the intra-abdominal findings to the cranial surgeries.

Cranial burr hole and subdural hematoma

Why did our subject merit a burr hole surgery, which was subsequently covered with a titanium alloy metallic cover? Gravity-assisted overdrainage of VP shunts, especially during the standing position, the so-called “siphon-effect,” is a well-documented problem with pressure-regulated VP shunts that was employed in our subject [2,6-8,16,17]. This could very well have resulted in SDH in our subject, necessitating the burr hole surgery for evacuation of the hematoma, and the subsequent implant of a titanium alloy burr hole cover [3]. While overdrainage is more often associated with SDH, underdrainage may also produce SDH, apart from other complications and manifestations. It may also be noted that SDH can also lead to shunt malfunction [2,6-8,16,17].

**Figure 15:**
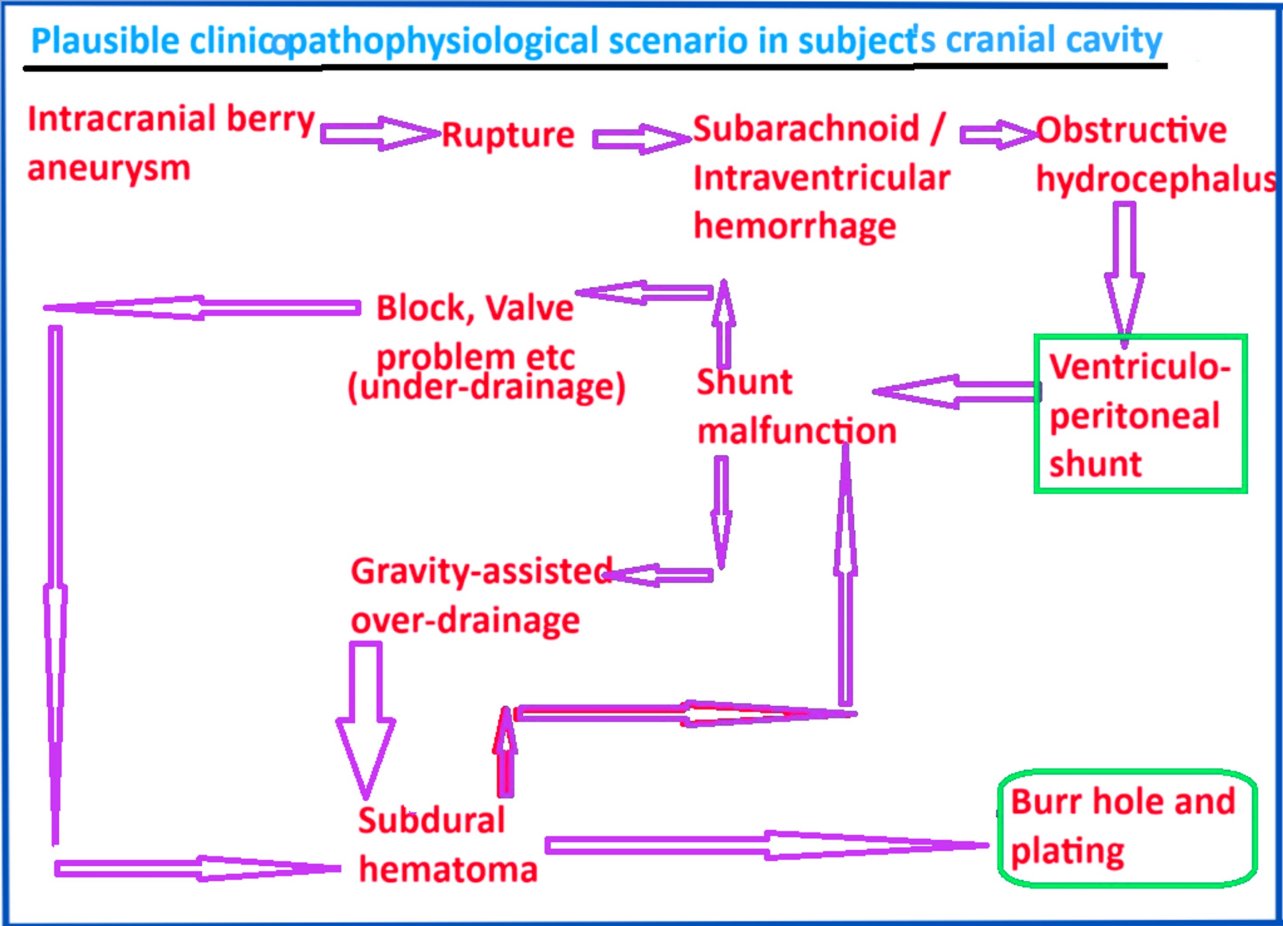
Schematic flow diagram demonstrating the postulated clinico-pathophysiological scenario in our subject to explain the intracranial findings. Ruptured intracranial aneurysm may necessitate VP shunt. Shunt malfunction could lead to SDH, indicating the need for burr hole surgery. SDH per se may also lead to shunt malfunction. Image created by the prinicipal author, assisted by Ms. Pritam Shahukar of All Saints University School of Medicine. It was created on MS Paint and enhanced using the Windows 11 Photos App. VP = ventriculoperitoneal; SDH = subdural hematoma

The possible sequence of clinico-pathophysiological events originating from aneurysmal SAH, leading to VP shunt implantation, and culminating in a burr hole surgery for SDH is summarized in the schematic flow diagram in Figure 15.

Positive unintended consequences in medical education

This case report establishes the positive effects of the law of unintended consequences when it comes to medical education [9]. While performing cadaveric dissections in our medical institution for anatomy education, we incidentally encountered a rare syndrome and three surgeries in the same subject. From the perspective of medical teaching, this was the positive effect of the unintended consequence alluded to earlier. Instead of merely restricting our teaching to Gross Anatomy for MD-1 students, we incorporated these incidental findings in the teaching curriculum of higher-level medical students, and even among surgical residents. The primary author of this paper has received numerous personal feedback on his cadaveric surgical dissection videos, from not only medical students but also medical graduates preparing for ECFMG, FRCS, etc., and surgical residents, testifying to the veracity of the statements in this case report. As holistic clinicians, while trying to establish clinico-pathophysiological correlations between these findings in the cadaveric subjects, it provided an opportunity to exercise our clinical acumen.

## Conclusions

Duodenal diverticular disease is closely linked to biliary lithiasis, with or without pancreatic involvement. Many of them may be missed cases of Lemmel syndrome, which may be unmasked by abdominal imaging techniques, notably ERCP, coupled with a high degree of clinical suspicion. Elderly females with aneurysmal SAH often require shunt surgery for alleviation of the increased ICP. Despite recent advances in shunt technology, shunt malfunction still constitutes one of the main complications. One such notable complication is SDH, which requires its own separate surgical intervention. Finally, anatomical dissection of cadavers should be performed with an open-minded approach toward the existence of unexpected pathologies in the subject. If such are found, they should be incorporated in higher-level medical teaching to provide a well-rounded and comprehensive clinico-pathophysiologic approach to medical education.
